# Analysis of risk factors for mental health problems of inpatients with chronic liver disease and nursing strategies: A single center descriptive study

**DOI:** 10.1002/brb3.2406

**Published:** 2021-11-11

**Authors:** Zhu Qin, Yannan Shen, Yuanhao Wu, Haicheng Tang, Lin Zhang

**Affiliations:** ^1^ Department of Hepatology Shanghai Public Health Clinical Center Fudan University Shanghai China; ^2^ Department of Critical Care Medicine Shanghai Public Health Clinical Center Fudan University Shanghai China; ^3^ Department of Nursing Shanghai Public Health Clinical Center Fudan University Shanghai China

**Keywords:** anxiety, chronic liver disease, depression, influencing factors, mental health, nursing

## Abstract

The number of patients with chronic liver disease (CLD) is large. The social and economic burdens due to CLD have increased. The mental health problems of patients with CLD are prominent and deserve our attention and care. This study analyzed 320 patients with CLD who were hospitalized between January 2018 and January 2020. Questionnaire surveys were used to assess mental health status, including the Self‐Rating Anxiety Scale (SAS), Self‐Rating Depression Scale (SDS), and Symptom Checklist‐90 (SCL‐90). At the same time, basic data and potential related factors were collected. Data were analyzed using descriptive statistics and logistic regression. Among the 320 patients with CLD, 240 (75%) had mental health problems; among the total patients, education levels, occupations, course of disease, annual hospitalizations, complications, and nursing satisfaction were significantly different between the two groups (*p* < .05). The education levels and occupations of the group without mental health problems were significantly different within the group (*p* < .05). The SCL‐90 found that the four factors with the highest scores were anxiety (ANX: 33.3%), depression (DEPR: 20.4%), somatization (SOM: 12.9%), and sleep and diet (SD: 9.6%). Logistic regression analysis showed that education levels, course of disease, annual hospitalizations, complications, and nursing satisfaction levels were independent risk factors for the mental health of patients with CLD. Model fitness was checked using the Hosmer–Lemeshow test. The receiver operating characteristic (ROC) curve showed that the area under the curve was 0.84. Patients with CLD have prominent mental health problems and experience many risk factors. It is necessary to adopt individualized psychological interventions and care to improve the quality of life of these patients.

## 1 BACKGROUND

The liver is the largest solid organ of the human body. Chronic liver disease (CLD) is a common cause of premature death. CLD is usually discovered late, and interventions are ineffective, resulting in a very high morbidity and mortality rate. According to data from the World Health Organization, hepatitis B virus is prevalent worldwide. Approximately 2 billion people in the world have been infected by the hepatitis B virus, and approximately 240 million of them have become chronically infected. The proportion of infection in different countries and different regions is quite different (Ott et al., 2012). According to the 2006 hepatitis B epidemiological survey report in China, the positive rate of Hepatitis B surface antigen (HBsAg) among people under 60 years old was 7.18% (Liang et al., 2009). Based on this, China has approximately 93 million people with chronic hepatitis B infection, of which 20 million are chronic hepatitis B patients (Lozano et al., 2012). The hepatitis C virus is also widespread. The total number of chronic hepatitis C virus infections in the world is approximately 130−170 million people, accounting for 2.8% of the world's total population. Approximately 4 million people are newly diagnosed with a hepatitis C virus infection each year. The possibility of developing chronic hepatitis after being infected with the hepatitis C virus is 75%−85%. In addition, nonviral CLDs also include cholestatic, alcoholic, fatty liver, and autoimmune liver diseases. Approximately 650,000 people worldwide die each year due to liver failure, cirrhosis, or liver cancer caused by CLD (Lozano et al., 2012).

The mental health of patients with CLD is easily overlooked during treatment, and psychological problems can affect the treatment effect and the outcome of the disease. At present, there are few studies on the psychological problems of patients with CLD at home and abroad. Patients with CLD experience repeated hospitalizations, poor treatment effects, and death due to complications, which can easily lead to psychological problems in patients, even if there are no complications. A variety of psychological and social problems can exist under these circumstances (Spiegel et al., 2007). Studies have shown that patients with severe CLD (cirrhosis and liver failure) have a higher degree of depression, and the degree of anxiety is not significantly abnormal compared with that of patients with hepatitis (Duan et al., 2012). The same research has proven that untreated patients with chronic hepatitis C also have various psychological problems, such as depression (Erim et al., 2010; Schaefer et al., 2012). Anxiety is common in patients with CLD. Seventy‐five percent of the patients had obvious anxiety after investigation, and 19% of them had anxiety and fear at the same time (Lopez‐Navas et al., 2013). Anxiety and fear not only affect treatment but also can harm society. Our hospital is a designated hospital for infectious diseases in Shanghai. This designated hospital treats a large number of patients with CLD every year. This study aimed to determine the risk factors affecting the mental health of patients with CLD through a mental health survey and to provide patients individualized treatment for these risk factors. Psychological interventions and nursing care can increase patients' confidence in overcoming the disease and achieve better clinical treatment results.

## 2 MATERIALS AND METHODS

### 2.1 Study design and sample

A total of 362 patients were hospitalized in the liver disease department of the hospital from January 2018 to January 2020, and 320 people completed the investigation. The diagnosis of CLD was in compliance with the clinical diagnosis and treatment standards. Exclusion criteria were as follows: hepatic coma, acute gastrointestinal bleeding, initial mental illness, and refusal to participate. According to their Self‐Rating Anxiety Scale (SAS) and Self‐Rating Depression Scale (SDS) scores, patients were divided into two groups. Patients with SAS and SDS scores less than 50 points were included in the healthy group (80 cases), and patients with SAS and SDS scores that were more than 50 points were included in the nonhealthy group (240 cases). The healthy group comprised 52 males and 28 females, aged 28−69 years, with an average age of 58.08 ± 8.32 years. In the nonhealthy group, there were 146 males and 94 females, aged 30−71 years, with an average age of 60.05 ± 5.35. There was no significant difference in age or sex between the two groups of patients (*p* > .05), and the groups were comparable.

### 2.2 Data collection

The demographic characteristics of the collected patients included occupational characteristics, age, sex, education level, marital status, occupation, course of disease, annual hospitalizations, aetiology, type of medical insurance, complications, and nursing satisfaction. This research was conducted in accordance with the Declaration of Helsinki. The data are accurate and reliable. This study was approved by the ethical review committee of the hospital. All patients provided informed consent. The questionnaire was anonymous and returned after the study.

#### 2.2.1 Self‐Rating Depression Scale

The SDS consists of 20 items and is divided into a four‐level self‐rating scale. It is easy to use and can fairly intuitively reflect the subjective feelings of depressed patients and their changes during treatment. If it is a forward scoring question, it will be rated as 1, 2, 3, and 4 points in turn; for a reverse scoring question, it will be rated as 4, 3, 2, and 1. After the evaluation is over, add up the scores of the 20 items to get the total score, and then multiply the score by 1.25, and take the integer part to get the standard score. The score ranges from 25 to 100. Index scores below 49 indicate no depression, 50–59 indicate mild to moderate depression, 60–69 indicate moderate to severe depression, and scores above 70 indicate severe depression (Zung, 1971).

#### 2.2.2 Self‐Rating Anxiety Scale

The SAS is a 20‐item self‐rating questionnaire used to assess anxiety‐related symptoms. Using a four‐level score, the frequency of symptoms defined by the main assessment items is: “1” no or very little time, “2” a small part of time, “3” a considerable amount of time, and “4” is extremely large part or all of the time. Similar to SDS, the original score of this scale ranges from 20 to 80 points. When converted into index scores, less than 50 is the normal range, 50–59 is mild to moderate anxiety, 60–69 is moderate to severe anxiety, and greater than 70 is severe anxiety (Dunstan & Scott, 2018; X. Wang et al., 1999).

#### 2.2.3 Symptom Checklist‐90

It is used to assess a wide range of psychological problems and psychopathological symptoms and has proven to be a useful tool. The scale consists of 90 items in nine dimensions. Each item is scored on a scale of 1–5, indicating asymptomatic to severe symptoms. The main symptom dimensions evaluated are as follows: somatization (SOM), obsessive‐compulsive symptoms (OCS), interpersonal sensitivity (INTS), depression (DEPR), anxiety (ANX), hostility (HOS), phobia and anxiety (PHOA), paranoia idea (PARI), psychosis (PSY), and sleep and diet (SD). Ten factors are used to reflect the psychological symptoms in 10 aspects. The scores for each factor are as follows: 1–1.99 points indicate no mental health problems, 2–2.99 points indicate mild mental health problems, 3–3.99 points indicate moderate mental health problems, 4–4.99 points indicate severe mental health problems, and 5 points indicate serious mental health problem. According to the national norm, if the total score is more than 160 points, the number of positive entries is more than 43 points, or any factor score is more than 2 points, it is considered positive, and further examination is required (Liu et al., 2017).

#### 2.2.4 Quality control

All patients are given standardized treatment after admission and receive routine care. The content includes: (1) oral care: assist in oral care of patients to prevent oral ulcers or infection; (2) diet care: due to the long course of CLD and poor nutritional status, it needs to be formulated. A nutritious diet plan improves the patient's appetite while avoiding spicy foods. The questionnaire is an anonymous survey, completed independently by the patient on the day of admission or the next day, supervised and guided by two nurses, and returned to the patient after all is completed.

### 2.3 Statistical analysis

We used SPSS21.0 software to analyze the data. The measurement data was expressed as (*x ± s*), and the comparison between groups was made by *t*‐test; the count data were expressed by *n* (%) and the *χ^2^* test was used. The logistic regression analysis was used for multivariate analysis. Receiver operating characteristic (ROC) curves were created. *p* < .05 indicates that the difference is statistically significant.

## 3 RESULTS

### 3.1 Sample description

Analyzing the demographic characteristics of the two groups of patients, there was no statistical difference in the gender, age, marital status, etiology, and type of medical insurance between the two groups (*p* > .05, Table 1).

**TABLE 1:** Analyzing the demographic characteristics of the two groups of patients

Characteristics	All (*n* = 320)	Healthy group (*n* = 80)	Nonhealthy group (*n* = 240)	*X^2^*	*p*‐Value
Gender				0.442	.506
Male	198	52	146		
Female	122	28	94		
Age group				3.040	.081
<50 years	167	50	117		
>50 years	153	30	123		
Education level				21.629	.000
Junior high school	105	10	95		
High school	120	35	85		
University	95	35	60		
Marital status				0.629	.730
Unmarried	33	8	25		
Married	230	60	170		
Divorced/widowed	57	12	45		
Occupation				10.730	.005
Farmer	54	6	48		
Freelance	104	22	82		
Permanent job	162	52	110		
Course of disease				15.607	.000
10 years	147	52	95		
10 years	173	28	145		
Annual hospitalizations				26.054	.000
<5 times	153	58	95		
>5 times	167	22	145		
Etiology				0.322	.956
Viral	204	52	152		
Autoimmunity	46	10	36		
Cholestatic	38	10	28		
Alcoholic	32	8	24		
Type of medical insurance				5.662	.059
Rural health insurance	104	18	86		
Employee health insurance	175	48	127		
Business insurance	41	14	27		
Complications				24.986	.000
No	143	55	88		
Yes	177	25	152		
Nursing satisfaction				7.007	.008
Good	163	51	112		
Bad	157	29	128		

*Note*: *p* < .05 indicates that the difference is statistically significant.

### 3.2 The SAS and SDS assess the mental health of patients with CLD

Among the 320 patients with CLD, 240 had mental health problems, with an incidence rate of 75%. Further analysis of the factors affecting patients’ mental health found that education level, occupation, course of disease, annual hospitalizations, complications, and nursing satisfaction were significantly different between the groups (*p* < .05, Table 1). A total of 240 patients in the nonhealthy group were divided into mild, moderate, and severe groups according to their SAS and SDS scores. The items with significant differences were compared between the groups. The results showed that there were significant differences between the groups in education level and occupation (*p* < .05, Table 2), while the course of disease, annual hospitalizations, complications, and nursing satisfaction comparisons between the groups were not statistically significant (*p* > .05, Table 2), further indicating that education level and occupation not only affect the mental health of patients with CLD but are also related to its severity.

**TABLE 2:** Self‐Rating Anxiety Scale (SAS) and Self‐Rating Depression Scale (SDS) assess the mental health of patients with chronic liver disease (CLD)

		Nonhealthy group				
Characteristics	*n* = 240	Mild (50–59, *n* = 135)	Moderate (60–69, *n* = 85)	Severe (>70, *n* = 20)	*X^2^*	*p‐*Value
Education level						
Junior high school	95	45	38	12	6.624	.036
High school	85	45	35	5	2.438	.296
University	60	45	12	3	11.435	.003
Occupation						
Farmer	48	28	12	8	6.885	.032
Freelance	82	44	31	7	0.355	.837
Permanent job	110	63	42	5	3.973	.137
Course of disease						
<10 years	95	47	40	8	3.271	.195
>10 years	145	88	45	12		
Annual hospitalizations						
<5 times	95	55	33	7	0.272	.873
>5 times	145	80	52	13		
Complications						
No	88	50	34	4	2.807	.246
Yes	1522	85	51	16		
Nursing satisfaction						
Good	112	66	38	8	0.756	.685
Bad	128	69	47	12		

*Note*: *p* < .05 indicates that the difference is statistically significant.

### 3.3 The Symptom Checklist‐90 assesses the mental health of patients with CLD

As shown in Table 3, in this study, the statistical results of the Symptom Checklist‐90 (SCL‐90) on the mental health of patients in the nonhealthy group found that 42 patients scored higher than 160 points, accounting for 17.5% of the group, 98 patients had positive items higher than 43 points, accounting for 40.8% of the group, and 63 patients had factor scores higher than 2, accounting for 26.3% of the group. Among all the factors, the average scores were as follows: SOM was 1.99 ± 0.21, OCS was 1.78 ± 0.31, INTS was 1.85 ± 0.24, DEPR was 2.03 ± 0.32, ANX was 2.21 ± 0.25, HOS was 1.67 ± 0.25, PHOA was 1.46 ± 0.54, PARI was 1.57 ± 0.43, PSY was 1.43 ± 0.35, and SD was 1.87 ± 0.26. We found that the four factors with the highest scores were ANX (33.3%), DEPR (20.4%), SOM (12.9%), and SD (9.6%).

**TABLE 3:** Symptom Checklist‐90 (SCL‐90) assesses the mental health of patients with chronic liver disease (CLD)

		Nonhealthy group (*n* = 240)					
Symptom	*n*	Mild (*n* = 136)	Moderate (*n* = 84)	Severe (*n* = 17)	Serious (*n* = 3)	Average	%
SOM	31	16	13	2	0	1.99 ± 0.21	12.9
OCS	11	6	5	0	0	1.78 ± 0.31	4.6
INTS	21	11	8	2	0	1.85 ± 0.24	8.8
DEPR	49	32	13	2	2	2.03 ± 0.32	20.4
ANX	80	42	30	7	1	2.21 ± 0.25	33.3
HOS	9	7	2	0	0	1.67 ± 0.25	3.8
PHOA	5	5	0	0	0	1.46 ± 0.54	2.1
PARI	7	5	2	0	0	1.57 ± 0.43	2.9
PSY	4	3	1	0	0	1.43 ± 0.35	1.7
SD	23	9	10	4	0	1.87 ± 0.26	9.6

*Note*: 1–1.99 points indicate no mental health problems, 2–2.99 points indicate mild mental health problems, 3–3.99 points indicate moderate mental health problems, 4–4.99 points indicate severe mental health problems, and 5 points indicate serious mental health problem. Abbreviations: ANX, anxiety; DEPR, depression; HOS, hostility; INTS, interpersonal sensitivity; OCS, obsessive‐compulsive symptoms; PARI, paranoia idea; PHOA, phobia and anxiety; PSY, psychosis; SD, sleep and diet; SOM, somatization.

### 3.4 Independent risk factors affecting mental health of patients with CLD

**FIGURE 1:**
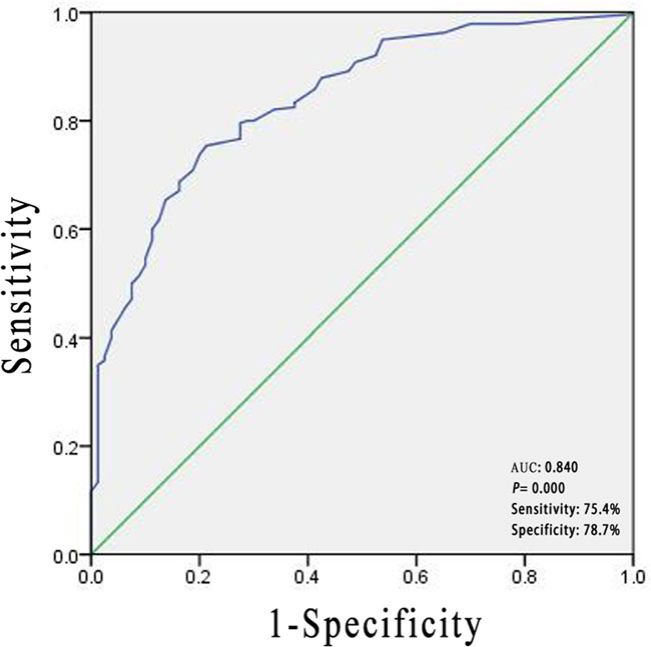
Receiver operating characteristic (ROC) curve analysis for the education level, course of disease, annual hospitalizations, complications, and nursing satisfaction value in the healthy group and nonhealthy group

Logistic regression analysis of the factors that may affect the mental health of patients with CLD showed that education level (*p* = .000, 95%confidence interval (CI) 0.197−0.525), course of disease (*p* = .002, 95%CI 1.470−5.541), annual hospitalizations (*p* = .000, 95%CI 1.801−6.766), complications (*p* = .000, 95%CI 2.130−7.848), and nursing satisfaction (*p* = .002, 95%CI 0.176−0.689) are independent risk factors for mental health problems in patients with CLD (Table 4). The Hosmer–Lemeshow test indicated a good fit of the model for predicting the variables. We combined five important variables (education level, course of disease, annual hospitalizations, complications, and nursing satisfaction) into the logistic regression to calculate the probability and made the ROC curve based on the probability we obtained. The ROC curve indicated an area under the curve (AUC) of 0.84. A cut‐off score of 0.542 yielded the maximum sum of sensitivity (75.4%) and specificity (78.7%) (Figure 1). Based on the results, the clinic can provide individualized psychological interventions and care for patients with CLD.

**TABLE 4:** Logistic regression analysis of factors that may affect the mental health of patients with chronic liver disease (CLD)

Characteristics	B	S.E.	Wals	*p*‐Value	Exp (B)	95% CI
Gender	0.688	0.464	2.199	.138	1.989	0.802–4.938
Age	0.315	0.345	0.835	.361	1.371	0.697–2.696
Education level	−1.135	0.251	20.513	.000	0.321	0.197–0.525
Marital status	0.118	0.310	0.145	.703	1.125	0.613–2.064
Occupation	−0.335	0.245	1.863	.172	0.715	0.442–1.157
Course of disease	1.049	0.339	9.597	.002	2.854	1.470–5.541
Annual hospitalizations	1.250	0.338	13.712	.000	3.491	1.801–6.766
Etiology	0.819	0.644	1.619	.203	2.269	0.642–8.012
Type of medical insurance	−0.429	0.253	2.873	.090	0.651	0.396–1.069
Complications	1.408	0.333	17.923	.000	4.089	2.130–7.848
Nursing satisfaction	−1.056	0.348	9.187	.002	0.348	0.176–0.689

*Note*: *p* < .05 indicates that the difference is statistically significant.

Abbreviations: CI, confidence internal; S.E., standard error.

## 4 DISCUSSION

CLD is a major public health problem. The number of patients with CLD is large, and the quality of life and work efficiency of these patients are reduced, which increases the social and economic burdens (Stepanova et al., 2017). In recent years, the clinical treatment of CLD has been in a stage of rapid development. In particular, there has been more in‐depth research on antiviral treatments. The mental health of patients with CLD is often neglected during treatment, and psychological problems will affect the treatment effect and disease outcome. The incidence of depression and anxiety in patients with chronic hepatitis is increasing year by year, and the incidence will increase as the course of the disease progresses. Failure to control the negative emotional state of patients with CLD in a timely manner can aggravate the negative response to treatment and the condition, causing CLD to recur without treatment for a long time, which seriously affects the treatment effect, reduces the patient's quality of life, and leads to psychological imbalance of the patient. Therefore, it is necessary to determine the risk factors for mental health problems of patients with CLD and conduct reasonable psychological treatments and nursing interventions in a timely manner for patients with CLD.

Epidemiological surveys conducted in several countries indicate that lower socioeconomic status (SES) is associated with increased liver‐related mortality (Crombie & Precious, 2011; Mackenbach et al., 2015; Najman et al., 2007; Tjepkema et al., 2012; Wong et al., 2002). In Denmark, low SES is associated with an increased risk of HCV infection. Once infected, patients with low SES have a higher mortality rate (Omland et al., 2013). Low social status is closely related to a low education level. Low education level (that is, not exceeding basic education) is an integral part of SES (Zung, 1971) and a reliable substitute indicator. Studies have found that in developed countries (Denniston et al., 2014; Meffre et al., 2010) and developing countries (Maan et al., 2014), a low education level is associated with a higher prevalence of HBV or HCV infection. Low education level is related to the severity of CLD. A low level of education is not a risk factor in itself, but it can be an indirect indicator of dangerous behaviors and habits that can increase the chances of subjects being exposed to the virus. This also could indicate a lack of knowledge and access to medical treatment, which may aggravate the prognosis of CLD (Stroffolini et al., 2020). Our research also found that occupation and education level affect the mental health of patients with CLD and affect the severity of mental health problems.

There are many reasons for the psychological problems of patients with CLD. Patients with CLD have a long course of disease, which can cause repeated acute exacerbations and hospitalizations during the course of the disease. This can affect the patient's work, leading to an inferiority complex and psychological shadow and indifferent interpersonal relationships, and some patients even lose their ability to work, leading to further increases in the economic burden. It is known that there will be complications in the course of CLD. The common complications are liver cancer (Uchida et al., 2020), liver fibrosis (Itakura et al., 2021), and upper gastrointestinal bleeding, and these complications are prone to causing liver failure, hepatic encephalopathy, and life‐threatening conditions. The patient suffers from the disease for a long time and eventually dies. These negative factors lead to mental health problems of patients with CLD. We used a more developed SAS and SDS to objectively evaluate the mental health of patients.

After a patient with CLD becomes sick, they lose interest in many things around them. If the patient looked at many trivial things before, they can develop depression, anxiety, eating and sleep disorders, etc. (Z. X. Wang et al., 2012). The SCL‐90 questionnaire test is the questionnaire survey that is used for evaluating comprehensive mental health and has good validity and reliability. In this study, the SCL‐90 questionnaire test in patients with CLD showed that patients with CLD do experience some mental health problems. The four factors with the highest scores were ANX (33.3%), DEPR (20.4%), SOM (12.9%), and SD (9.6%). Overall, patients with CLD have increasingly complex psychological problems, that are worthy of social attention.

In short, in addition to providing more professional treatments for patients with CLD, patients with mental health problems require more psychological counselling and care from our doctors and nurses. Interestingly, our research has found that nursing satisfaction also exists in patients with CLD. Nursing satisfaction was an independent risk factor for mental health problems mainly because we are a large specialist hospital with a shortage of nursing staff, extended working hours for nurses, and insufficient time to communicate with patients. At the same time, the professional skills of nurses need to be further improved, especially in psychology. Knowledge in this area must be enriched so that we can better and more accurately care for patients, form a precise and individualized nursing model, achieve satisfactory nursing results, reduce the physical and psychological pain of patients, and truly improve the quality of life of patients.

## 5 CONCLUSION

Patients with CLD have prominent mental health problems and experience many risk factors, such as education level, course of disease, annual hospitalizations, complications, and nursing satisfaction. It is necessary to adopt individualized psychological interventions and care to improve the quality of life of these patients.

## 6 LIMITATIONS

Several limitations should be considered in the present study, including the sample from a single hospital. In the future, research should be conducted in multiple hospitals to better generalize the findings and compare the differences. Finally, access to social and family support for CLD should also be provided.

## 7 RELEVANCE FOR CLINICAL PRACTICE

The results of this study provide information for patients with CLD who have mental health disorders during their hospitalization. This information may help doctors and nurses or hospital authorities take necessary measures to provide support and interventions for the mental health of patients with CLD who are more likely to develop psychological problems than other types of patients.

## CONFLICT OF INTEREST

The authors declare no conflict of interest.

## AUTHOR CONTRIBUTIONS

Zhu Qin designed the study, screened the literature, and drafted the manuscript. Yannan Shen and Yuanhao Wu collected the clinical data and processed statistical data. Haicheng Tang analyzed and interpreted the data and revised the manuscript. Lin Zhang designed, supervised, and revised the manuscript. All authors read and approved the final version of the manuscript.

### TRANSPARENT PEER REVIEW

The peer review history for this article is available at https://publons.com/publon/10.1002/brb3.2406

## Data Availability

The original source data and material will be available upon reasonable request.
